# Cardiorespiratory Improvements Achieved by American College of Sports Medicine’s Exercise Prescription Implemented on a Mobile App

**DOI:** 10.2196/mhealth.5518

**Published:** 2016-06-23

**Authors:** Gianluca Rospo, Viola Valsecchi, Alberto G Bonomi, Inge WJ Thomassen, Saskia van Dantzig, Antonio La Torre, Francesco Sartor

**Affiliations:** ^1^ Universita' degli Studi di Milano Milan Italy; ^2^ Philips Research Eindhoven Netherlands; ^3^ Technische Universiteit Eindhoven Eindhoven Netherlands

**Keywords:** cardio-respiratory fitness, ACSM guidelines, physical activity, mobile app

## Abstract

**Background:**

Strong evidence shows that an increase in cardiorespiratory fitness (CRF) and physical activity (PA) reduces cardiovascular disease risk.

**Objective:**

To test whether a scientifically endorsed program to increase CRF and PA, implemented on an easy-to-use, always-accessible mobile app would be effective in improving CRF.

**Methods:**

Of 63 healthy volunteers participating, 18 tested the user interface of the Cardio-Fitness App (CF-App); and 45 underwent a 2-week intervention period, of whom 33 eventually concluded it. These were assigned into three groups. The Step-based App (Step-App) group (n=8), followed 10,000 steps/day prescription, the CF-App group (n=13), and the Supervised Cardio-Fitness (Super-CF) group (n=12), both followed a heart rate (HR)-based program according to American College of Sports Medicine (ACSM) guidelines, but either implemented on the app, or at the gym, respectively. Participants were tested for CRF, PA, resting systolic and diastolic blood pressures (SBP, DBP), resting, exercise, and recovery HR.

**Results:**

CRF increased in all groups (+4.9%; *P*<.001). SBP decreased in all groups (-2.6 mm Hg; *P*=.03). DBP decrease was higher in the Super-CF group (-3.5 mm Hg) than in the Step-App group (-2.1 mm Hg; *P*<.001). Posttest exercise HR decreased in all groups (-3.4 bpm; *P*=.02). Posttest recovery HR was lower in the Super-CF group (-10.1 bpm) than in the other two groups (CF-App: -4.9 bpm, Step-App: -3.3 bpm; *P*<.001). The CF-App group, however, achieved these improvements with more training heart beats (*P*<.01).

**Conclusions:**

A 10,000 steps/day target-based app improved CRF similar to an ACSM guideline-based program whether it was implemented on a mobile app or in supervised gym sessions.

## Introduction

Low cardiorespiratory fitness (CRF) and physical activity (PA) have been shown to be two key independent risk factors for cardiovascular disease (CVD) and all-cause mortality [1-4]. These two are causally interconnected, although partially distinct [3]. High CRF is associated with higher habitual PA levels [5], and habitual PA seems to be the most important contributor to CRF [3,6]. Therefore, both CRF and PA should be fundamental elements of any health intervention program, along with other crucial elements, such as diet and smoke cessation.

Ten thousand steps a day is a well-accepted PA goal, which has shown benefits in improving people’s cardiovascular health [7]. The sustainability of a 10,000 steps/day target was evaluated in the whole Flemish community with a 1.5-year follow-up, showing an implementation rate of 58% among all contacted organizations, and that citizens aware of the 10,000 steps/day program were on average more active than those unaware [8]. As for other behavioral change programs, the likelihood of meeting the 10,000 steps/day goal depends also upon the intention to change and self-efficacy [9]. Yet, a program based on step counting, when the target is personalized rather than a fixed number for all subpopulations, would have the advantage to be compatible “with public health recognition that some physical activity is better than none“ [10]. Although this is a very clear and straightforward goal, rather easy to implement in the general population, a PA program based exclusively on step counting does not necessarily have to be the most efficient program to improve people’s cardiovascular health [11]. As pointed out by Tudor-Locke et al [10] 10,000 steps/day would be adequate for some people to accumulate 30 minutes of moderate to vigorous PA a day, and for others this would not be the case. Accordingly, the American College of Sports Medicine (ACSM) guidelines emphasize to engage in moderate and vigorous exercise intensity [5] in order to improve CRF. Training programs in line with these recommendations are usually designed based on target heart rate (HR), as a percentage of maximal HR or heart rate reserve (HRR) [5]. The efficacy of these new ACSM’s guidelines accounting for exercise intensity was confirmed by several investigations [12-14]. In particular, Huang et al [12] and Swain [14] identified moderate intensity from 66% to 73% HRR, 40 to 50 minutes per session, 3 to 4 times per week, for 9 months, optimal to improve CRF in healthy sedentary adults and safer than vigorous intensity exercise. Schoenbron et al [13] brought direct evidence that adhering to the 2008 PA guidelines was associated with a reduction in all-cause mortality. Nevertheless, HR-based programs may result less straightforward to implement in the general and patient population, because of issues such as, chronotropic incompetence, and HR-lowering medications, inaccurate prediction of maximal HR [15]; and more difficult to understand than number of steps/day [16].

Next, to the issue of what is the most effective training program to reduce CVDs risk, there is the problem of getting people to adhere to such a program. Low adherence to PA and exercise programs has been a known issue for decades [17]. Many people do not manage to make regular use of health clubs for various reasons, among which are distance and perceived lack of time. Indeed, health clubs subscribers are known to overestimate their future attendance [18]. Some independent predictors of adherence such as demographic, and general health are particularly hard to address [17,19]. However, some other common barriers such as program-related factors (eg, lack of personalization), [17,20] and environmental factors (eg, find the time and place) [17,21] could be addressed by an appropriate lifestyle proposition.

Recently, smart, wearable, and mobile technology has enabled a large number of health applications [22]. As it was indicated in a recent systematic review, smartphones are more and more often used to measure and promote PA; however, currently still with modest results [23]. It has been shown that Web-based apps to improve and self-monitor PA were well received by middle aged men as long as the information was delivered quickly and it was easy to use [24]. As a confirmation of this, connected monitoring tools, such as smartphones, pedometers, and blood pressure devices used to support an exercise intervention could be overwhelming to users if those are asked to process too much information [25].

Knight et al [22] reviewed 379 PA apps, finding that none of those apps included public health recommendations for aerobic physical activity. The use of smartphones presents an important opportunity in rolling out an endorsed program aimed at improving CRF, because of the numerous possibilities that these platforms can enable, such as, to give real-time feedback on people’s progress, to flexibly fit in people’s daily schedule, and to connect with social networks allowing for social support.

Therefore, the primary aim of this study was to test whether a scientifically endorsed program to increase CRF implemented in an easy to use, and always-accessible mobile app, would be effective in improving CRF. A secondary objective of this study was to investigate how a HR-based training would compare with a steps-based training in terms of CRF changes.

## Methods

### Participants and Study Design

In order to test our research hypotheses a three group, pre- and posttest, 2-week intervention study was designed. The study protocol was approved by the Internal Committee of Biomedical Experiments of Philips Research as well as the Departmental Ethics Committee of the Milan University according to the Declaration of Helsinki.

Sixty-three participants were included in this study. Eighteen took part in a user experience test of the app. Forty-five participants took part in the actual experimental intervention protocol. Those participants were recruited via posters and at the local health center. After first interest in the study, volunteers were informed about the study protocol via information letters, where inclusion and exclusion criteria were already mentioned. These criteria specified, among others, the absence of chronic health conditions, no cognitive impairments, a low cardiorespiratory fitness (<45 mL/kg/min), an age ranged between 20 and 55 years, and a body mass index (BMI) limit not exceeding 35 kg/m^2^. After signing the informed consent participants were screened for cardiovascular risk using the American Heart Association/ACSM Health/Fitness Facility Preparticipation Screening Questionnaire [26] and for PA readiness by means of the Physical Activity Readiness Questionnaire [27]. Participants were also asked to fill in a nonexercise (N-Ex) aerobic capacity questionnaire [28] to roughly estimate their initial fitness level. Self-efficacy to adhere to an exercise routine was evaluated via Bandura’s questionnaire [29].

**Figure 1:**
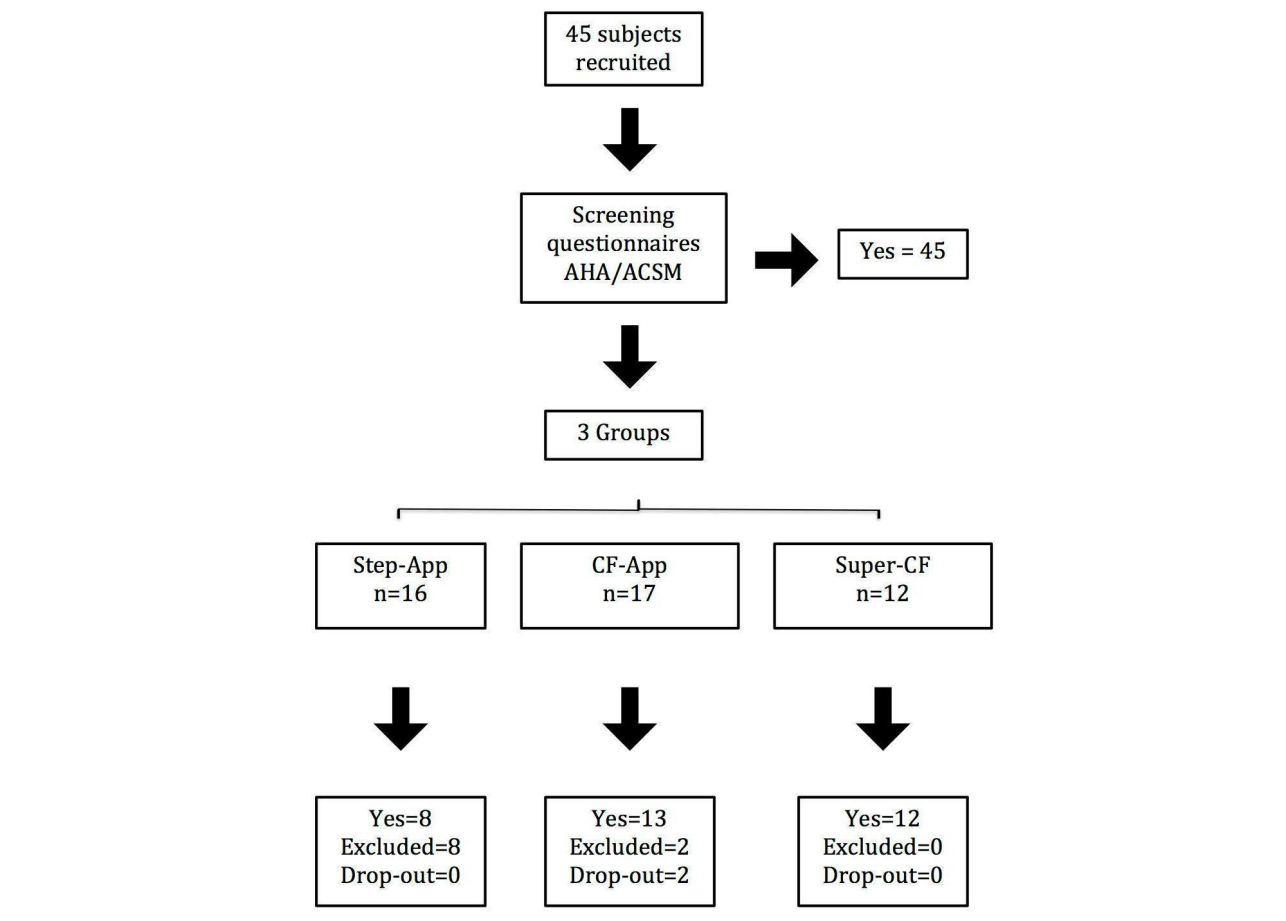
Study enrollment flow-chart. Step-App, 10,000 steps/day training plan provided by a mobile app; CF-App, ACSM guidelines-based cardio-fitness training plan provided by a mobile app; Super-CF, ACSM guidelines-based cardio-fitness training plan provided by a personal trainer.

The latter 45 participants were assigned into three groups: the Step Count App group (Step-App) (n=16), the Cardio Fitness App group (CF-App) (n=17), and the Supervised Cardio Fitness group (Super-CF) (n=12). For logistic reasons only two groups, the Step-App group and the CF-App group, could be randomized. This is because the participants included in the Super-CF group, which was included as a training intervention quality check, had to be living in the vicinity of the fitness center used for the supervised exercise intervention. This has resulted in an inhomogeneity at baseline between this group and the other two. Participant characteristics at baseline are reported in Table 1. After a baseline CRF evaluation, 10 participants were excluded from the study because having a treadmill test estimated VO_2_ max > 45 mL/kg/min (Figure 1).

**Table 1:** Participant characteristics.

	Step-App^a^ group (n=8)	Super-CF^b^ group (n=12)	CF-App^c^ group(n=13)
	Mean ± standard deviation	Mean ± standard deviation	Mean ± standard deviation
Male/female	3/5	5/7	5/8
Age, years	40 ± 10	45 ± 3	42 ± 6
Height, m	1.69 ± 0.03	1.69 ± 0.12	1.71 ± 0.09
Weight, kg	68.20 ± 11.80	78.10 ± 19.10	81.60 ± 10.10
BMI, kg/m^2^	23.70 ± 3.53	27 ± 4.70	26.60 ± 1.43
SBP^d^, mm Hg	124 ± 11.60	132 ± 11.14	129 ± 16.80
DBP^e^, mm Hg	76.90 ± 8.06^f^	87.40 ± 5.68^f^	83.70 ± 9.60
N-Ex^g^ questionnaire	3.50 ± 2.27	0.58 ± 0.79	3.08 ± 2.27
Self-efficacy questionnaire^h^	57.70 ± 3.31	44.20 ± 9.60	61.60 ± 13.98
Estimated VO_2_ max, mL/kg/min^i^	36.8 ± 4.90^j^	26.9 ± 5.60^j^	31.70 ± 6.40

^a^Step count app group.

^b^Supervised cardio fitness group.

^c^Cardio fitness app group.

^d^Systolic blood pressure.

^e^Diastolic blood pressure.

^f^Significant differences at the baseline between Step-App and Super-CF groups are *P*<.01.

^g^Nonexercise aerobic capacity questionnaire (where 0 is inactive and 7 is very active).

^h^The self-efficacy questionnaire is on a 0 to 100 scale.

^i^Self-paced treadmill walk test was used to estimate VO_2_ max.

^j^Significant differences at the baseline between Step-App and Super-CF groups are *P*<0.05.

### Testing Sessions

**Figure 2:**
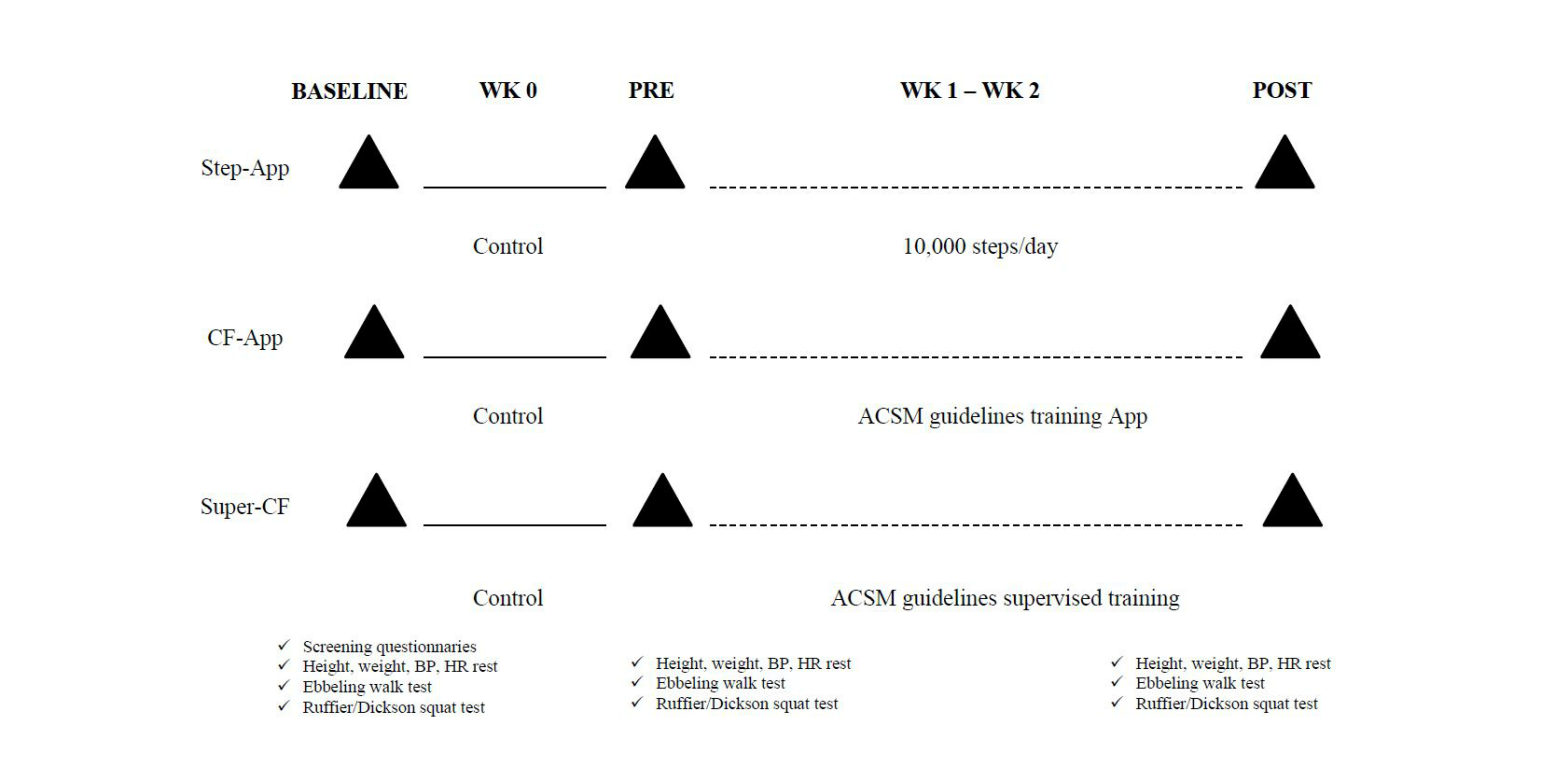
Study protocol outline.

Participants were asked to visit our laboratories on three different occasions; for the baseline tests, the pretests, which occurred 1 week after the baseline tests, and the posttests, which took place immediately after the 2-week intervention. The design of the study is depicted in Figure 2. In order to characterize the population tested in this study and to confirm the self-reported inclusion parameters, height and weight, and therefore BMI, resting blood pressure and CRF were assessed. After the anthropometric measurements participants were asked to wear a HR chest strap monitoring device throughout the entire testing session. The resting HR was recorded while the participant was seated in a quiet and dimmed light environment for 3 minutes. At the end of this period the resting blood pressure was measured.

Two submaximal exercise tests were then conducted. The Ruffier-Dickson squat test [30] and the Ebbeling single-stage treadmill walk test [31]. The Ruffier-Dickson squat test consisted of a 45- second paced (40 bends/min) squatting exercise, followed by a 3-minute recovery period. Parameters evaluated were resting HR prior to the squatting exercise, HR at the end of the exercise, and recovery HR after 1 and 3 minutes. Moreover, the Ruffier-Dickson Index (RDI) was calculated according to the following equation:

RDI=(P_1_-70)+2(P_2_-P_0_)/10 (1)

where P_0_ is 15-seconds mean resting HR, P_1_ is the maximum HR recorded during the first 15 seconds of recovery, and P_2_ is the 15-seconds mean after the first minute of recovery (the period from 1 minute and 00 seconds to 1 minute and 15 seconds) [30].

The Ebbeling test consisted of a 4-minute walking session at an adequate speed so that the participant’s HR would be between 50% and 75% of the estimated max HR (220 minus Age), followed by a 4 to 5 minutes session at a 5% incline at the same speed [31]. If HR between the seventh and the eighth minute did not differ more than 6 bpm, the test was ended, otherwise the test was continued for 1 more minute. VO_2_ max was estimated according to the following equation:

VO_2_ max=15.1+(21.8 · Speed)-(0.327 · HR)-(0.263 · Speed · Age)+(0.00504 · HR · Age)+(5.98 · Gender) (2)

where treadmill speed was in miles per hour, HR in beats per minute, age in years, and gender was 0 for females and 1 for males. Except for the screening questionnaires and the height measurement, all other physical tests performed at baseline, were being repeated at pretest and after the 2-week intervention.

### Training Programs

Throughout the entire study period participants were asked to wear two HR monitors, a chest strap–based one, the same as mentioned above, and a wrist mounted optical sensor, validated by Valenti and Westerterp [32], which was able to send second by second HR data to a smartphone via Bluetooth connection. The smartphones were provided by the research team for the entire duration of the study. Participants’ steps were assessed by a pedometer. This was clipped to the right pocket of the trousers or in absence of such pockets to the waist belt. All monitoring devices were taken off during the night and when the participants were showering or taking a bath.

During the pretest visit, participants received instructions about the training that they would follow during the two intervention weeks. The Step-App group was asked to complete 10,000 steps per day. Participants could access feedback on their progress via the device and via a standard mobile app, only during week 1 and 2, and not during the control week. No specific instructions were given to this group on how to achieve their goal. The pedometer has a display were steps could be read upon request, and it can be synchronized via Bluetooth connection directly with its dedicated mobile app. The Fitbit app did not provide any strategy on how to achieve the 10,000 steps/day target neither gave reminders.

**Figure 3:**
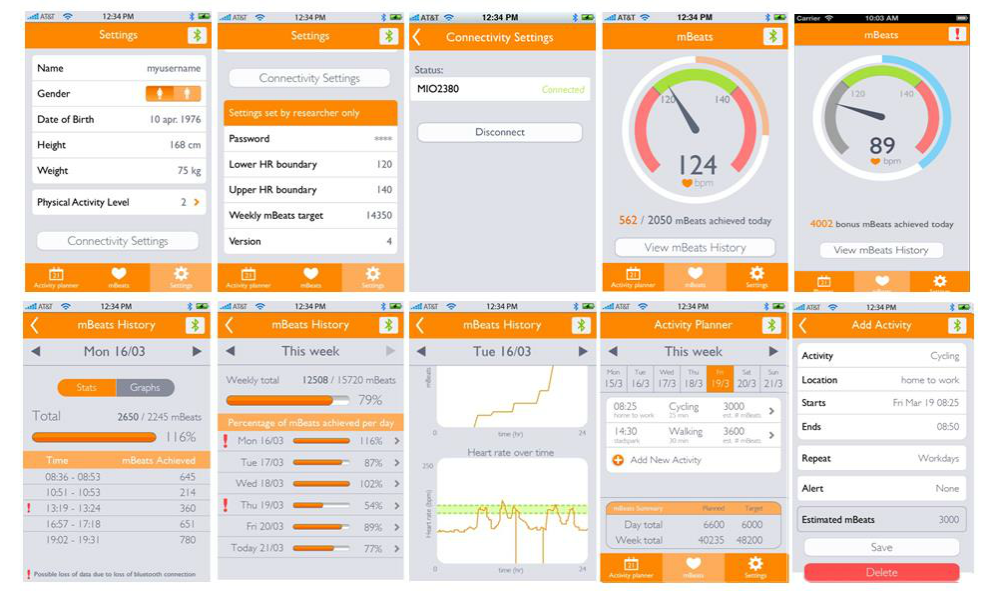
Multiple screenshots of the Cardio Fitness mobile app used in this study.

The CF-App and Super-CF groups were asked to follow an intensity training based on the guidelines of the ACSM [5]. The main difference between these two groups was that the CF-App group received feedback on their progress via our CF-App (Figure 3), whereas the Super-CF group was asked to attend training sessions three or four times a week, depending on their starting CRF level, and received personal feedback only during those supervised sessions.

The training programs for the CF-App and the Super-CF groups were designed according to the recommended frequency, intensity, time, and type framework outlined in Table 7.4 of the ACSM’s guidelines for exercise testing prescription consistent with the United States Department of Health and Human Services Physical Activity Guidelines for Americans [5]. Physical fitness classification was done according to Table 4.8 of the above mentioned ACSM’s guidelines [5]. Because the guidelines provide ranges for frequency, intensity, and duration, we have selected the minimum value of the range as a guideline on which to give feedback.

For the CF-App group, the daily training target was visualized based on the concept of the Training Impulse method by Banister and Calvert [33]. The idea behind this visualization was to have a simple metric similar to steps per day that the participants could immediately understand.

This training monitoring metric was called “mBeats” and consisted of the number of heart beats in a personalized heart rate zone. This mBeats score was calculated over the week as target HR (bpm) × session duration (minutes) × the training frequency. Target HR was defined according to Box 7.2 of the ACSM guidelines [5], that is,

Target HR=[(HR_max_–HR_rest_)] · %intensity desired]+HR_rest_ (3)

where the desired intensity is determined according to the baseline fitness level, corrected for age and gender. For instance participants with a target HR=120 bpm (eg, 30% of HRR) and classified as sedentary would receive a training frequency of three times a week, and a training session duration of 30 minutes, making a weekly mBeats target equal to target HR × session duration × frequency per week of 10,800 mBeats. The week was divided into training days and resting days. In this example, a training day would have a daily target of 3600 mBeats, while a resting day would have a target of 0 mBeats. The daily targets are not fixed, but depend on the remaining mBeats for the week. In other words, if the weekly target mBeats was 10,800 and on the first training day 7000 mBeats were already achieved, the daily target on the second training day would have been 1900 mBeats (the remaining 3800 mBeats divided by 2 remaining training days of the week).

The participants in the Super-CF group were instructed to visit the gym three to four times per week according to their training program consistent with the ACSM guidelines described above. During the training session at the fitness center participants were supervised and motivated by an experienced personal trainer. They were also asked to wear the HR monitors and step counters for the whole day during the entire intervention period without a specific goal.

### Cardio Fitness Mobile App and Its User Experience

A mobile app was specifically designed for the purpose of this study. Main features included in this first prototype of the CF-App were a HR feedback element, daily and weekly mBeats targets, an activity planner based on the theory of implementation intentions [34], and progress toward the mBeats targets. Each of the screens was designed using Adobe Illustrator CS6, taking the Philips Communication guidelines and the Apple Human Interface Guidelines into account. Intuitiveness of the interface was tested by paper prototyping. To increase intuitiveness and simplicity, the design of the app elements was derived from standard Apple iOS interface elements. The app was compatible with Apple iPhone 5, with a screen resolution of 3.5 inch; 960 × 640 pixels. The main screen of the app showed the training HR zone, current HR, target amount of mBeats for that day, and progress toward this target. The main screen also showed a button ‘view mBeats history’. This button led to a weekly overview of the percentage of achieved mBeats per day and per week (Figure 3).

Our App included a planning option, in which the participants could decide how to distribute their training days according to their own private schedule (Figure 3). This planning option also forecasted the number of mBeats that would be achieved by following one’s personal plan. This feature was intended to help the participants understand beforehand whether their plan would be sufficient to reach their mBeats target. The CF-App was flexible to plan changes allowing the user to collect bonus points (ie, mBeats) during a resting day (Figure 3). Finally, the home screen of the app showed the current HR and whether this would be in the mBeats zone; the goal was displayed in a very simple fashion as an odometer, where achieved mBeats as a proportion of the day target was visualized in a filling ring (Figure 3). The participants in the CF-App group were instructed how to use the CF-App during the second visit (pretest).

### User Experience Pilot Test

User interaction experience was tested during a 3-week pilot test by 18 healthy adults (age: 26-50 years, BMI: 18-25 kg/m2). The test started with a baseline week followed by two intervention weeks. During the baseline week, participants were instructed to wear the HR monitor and keep the app running, in order to collect physical activity data. All functionalities of the app were disabled and participants were asked to be as physically active as usual. After the baseline week, the 2-week intervention period started in which participants were coached to achieve their daily and weekly mBeats targets. At the end of the study, a one-on-one, semistructured interview was conducted to discuss usability and user experiences in depth. In addition, usability was measured with the Computer System Usability Questionnaire (CSUQ) [35]. This questionnaire consists of 19 items, categorized into three variables; system usefulness (items 1-8), information quality (items 9-15), and interface quality (items 16-19). In addition, an overall usability score is computed-based on all items.

### Statistical Analysis

The Statistical analysis was performed using the Statistical Package for Social Sciences (version 21) and the level of statistical significance was set at 0.05. Data were presented as means ± standard deviations unless otherwise indicated. Dependent variables were analyzed with a two-way, repeated, measures-mixed analysis of the variance (ANOVA), where the two factors were: time (pre- and post-intervention) and group (Step-App, CF-App, Super-CF). Because only the Step-App and the CF-App groups were randomized, a two-way, repeated measures ANOVA was performed also only on those two groups. If there had been violations of the sphericity, the corrections of Green-house Geisser if ɛ<0.75 and Huynh-Feldt if ɛ>0.75 were applied. The significant interactions were followed by post-hoc Tukey test. Pearson correlation coefficients were calculated between RDI and estimated VO_2_ max.

## Results

### Cardio-Fitness App Usability

Overall, participants were very positive about the potential of the CF-App and the concept of collecting heart beats to increase PA. Nonetheless, the pilot test revealed some points for improvement for the app. Most participants stated that their target zone was too narrow, and for some participants the zone was too high. Based on this feedback, the target HR zone was widened in the intervention study. Moreover, more features and functionality could be added to make the CF-App useful for people with various health goals. The current app is specifically designed to improve CRF, but over half of the participants stated they would want more information about the effects of all activities, including activities that are in a different HR zone. Furthermore, they wanted to be able to set their own exercise goals, to, for example, maintaining health or losing weight.

Other than many other popular fitness apps, the CF-App stimulates users to achieve a weekly exercise target, rather than daily targets. The majority of participants (15/18, 83%) were in favor of a weekly target, as opposed to a daily target, because the amount of exercise they do is not the same for every day, but is fairly similar every week. Five of them stated that although they preferred a weekly target, they would be okay with having a daily target, as long as the daily target is integrated with what they have planned in their activity planner. Only three participants were in favor of the daily target because it would motivate them to get enough exercise on a daily basis. Overall usability from the CSUQ was toward the positive end of the scale. On a scale from 1 to 7, the participants rated usability 4.46 ± 1.46, quality of the interface 4.96 ± 1.26, system usefulness 4.72 ± 1.34, and information quality 4.21 ± 1.28.

The ratings on the CSUQ questionnaire were in line with the qualitative findings obtained during the interviews. Ratings on the three variables were on the positive side of the scale, confirming the potential of the app. However, with an average rating of 4.5 on a 7-point scale, usability is not rated extremely high. This can mostly be accounted to some technical issues and the limited functionality of our first prototype. Moreover, participants identified missing elements that would make the app better, more interesting, and easier to interpret. Half of the participants explicitly stated they would add more parameters, such as speed and distance. This would make it easier for them to relate the mBeats to something they are already familiar with. Ten participants would like to add global positioning system tracking, or at least link the timestamps in the history view to a location, because it could give them insight in the effects of certain routes on their exercise performance.

A majority of the participants mentioned that they would like advisory or motivational messages in the app. These could be tips on what kind of activities they could do to reach their target (8 participants), or information on how they are doing during exercising (6 participants). In addition, six participants mentioned they want to get messages about their achievements so far, like how many beats they still need to achieve, and another six participants wanted motivational messages such as ‘good job’ or ‘you have been idle for a while, isn’t it getting time to go for a jog?’

Some participants did not fully understand the mBeats concept. They would need more education on advantages of the concept and measuring heart rate. Furthermore, they would need support in interpreting their data, to gain better understanding of their performance.

Because people were uncertain about their current level of fitness and how to improve or maintain it, they would want the app to objectively assess their current fitness level and use this information to create a personalized training program, with feasible goals and reliable, and accurate HR zones.

### Weight and Blood Pressure

**Figure 4:**
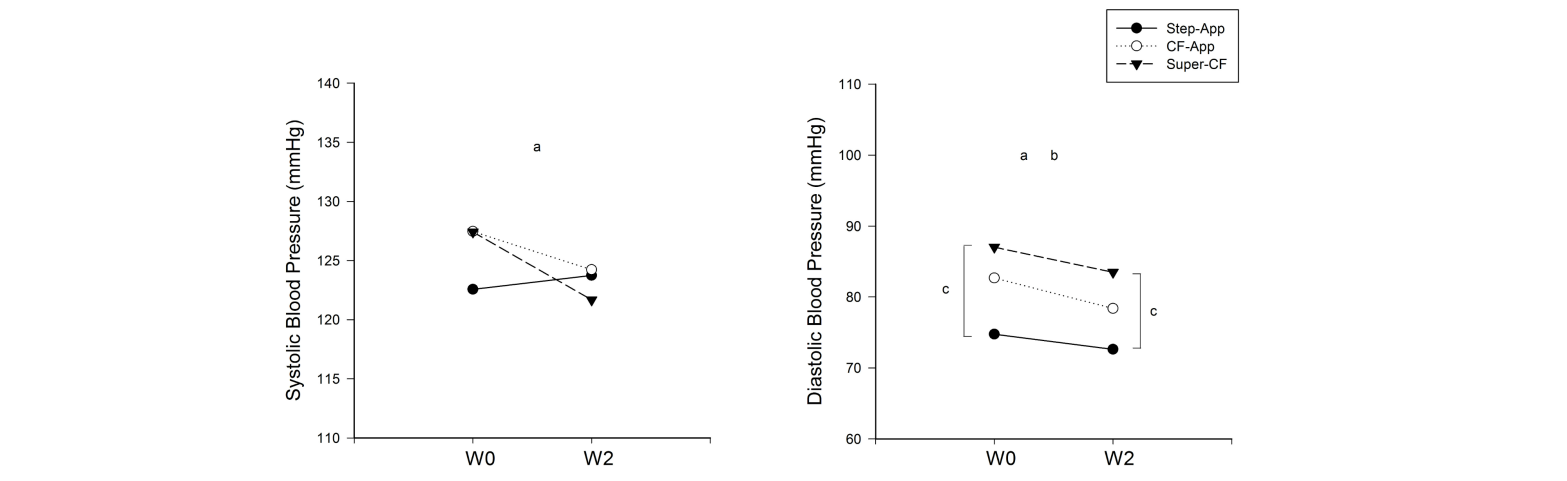
Baseline (W0) and 2 weeks (WL2) systolic and diastolic blood pressure changes induced by the interventions. Step-APP, 10,000 steps/day training plan provided by a smartphone application; CF-App, ACSM guidelines-based cardio-fitness training plan provided by a smartphone application; Super-CF, ACSM guidelines-based cardio-fitness training plan provided by a personal trainer. a, Significant main effect of time. b, Significant main effect of group. c, Significant difference between Step-App group and Super-CF group.

There were no significant time × group interactions (F(1;30)=0.466, *P*=.63), nor main effect of time (F(1;30)=0.189, *P*=.67) and of group (F(1;30)=2.153, *P*=.13) in body weight. No time × group interaction was present for systolic (F(1;30)=2.169, *P*=.08) and diastolic (F(1;30)=0.426, *P*=.66) blood pressure. Although, a significant main effect of time was observed in both systolic blood pressure (SBP) (F(1;30)=4.946, *P*=.03; Step-App: +1.19 mm Hg; CF-App: -3.23 mm Hg; Super-CF: -5.75 mm Hg) and diastolic blood pressure (DBP) (F(1;30)=12.585, *P*<.001; Step-App: -2.12 mm Hg; CF-App: -4.31 mm Hg; Super-CF: -3.54); only the DBP showed a significant main effect of group (F(1;30)=5.765, *P*<.01). Following-up these significant differences between groups it was observed that the DBP in the Step-App group was lower than in the Super-CF groups at baseline (*P*<.01) as well as at week 2 (*P*<.001) (Figure 4). When only Step-App and CF-App were compared, a main effect of group for body weight, CF-App being heavier (F(1;19)=7.345, *P*<.05), and a main effect of time for DBP (F(1;19)=6.829, *P*<.05) were found.

### Estimated Maximal Oxygen Uptake, Step Counts, and mBeats

**Figure 5:**
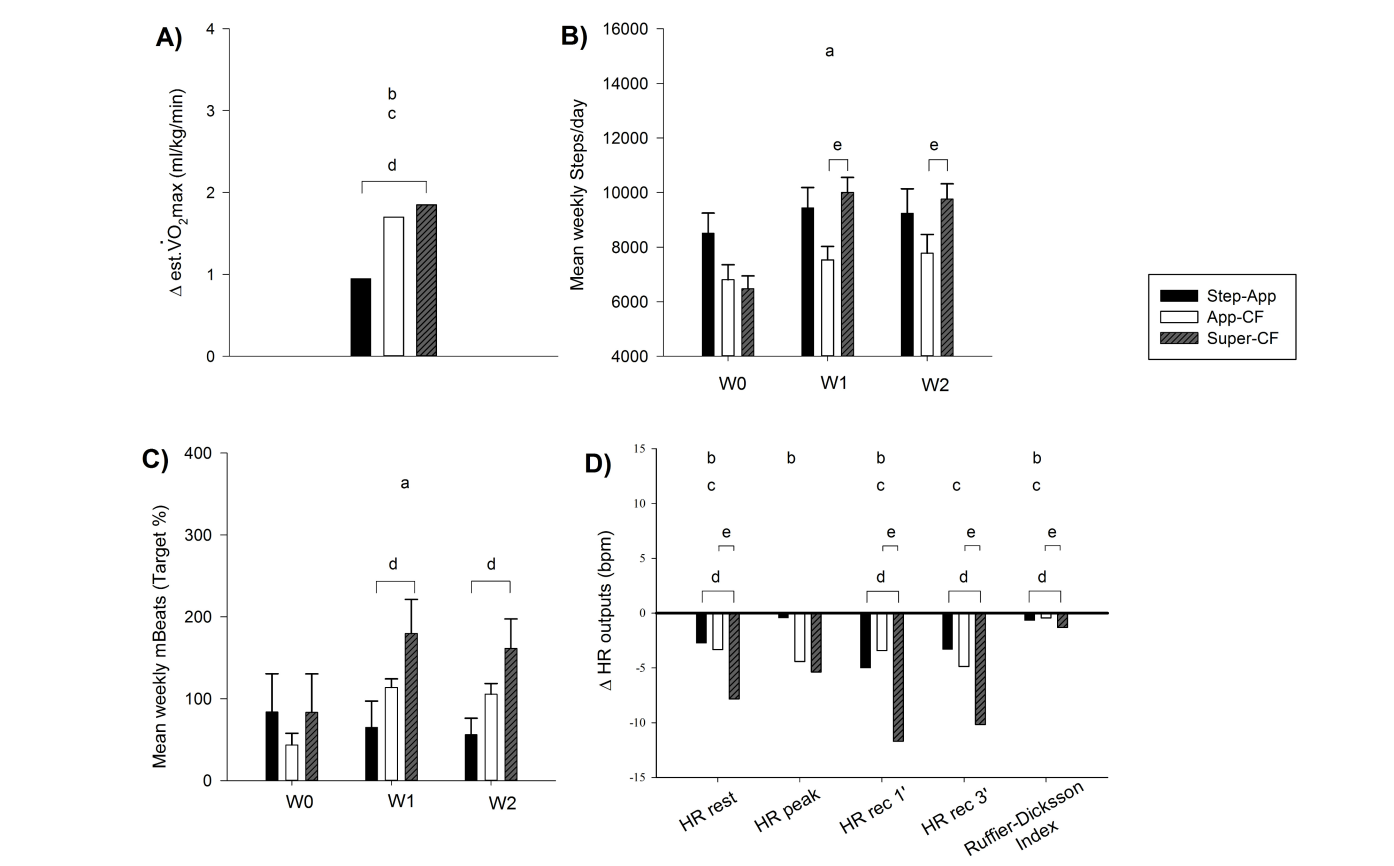
A) VO_2_ max week 2 – baseline deltas. B) Mean weekly steps showed for the baseline week 0 (W0), and the two intervention weeks, week 1 (W1), and week 2 (W2). C) Mean weekly mBeats expressed as percentage of target mBeats (for the definition of mBeats see methods section). D) Week 2 - baseline Heart rate (HR) deltas at rest, for the maximal recoded during a squat exercise test (peak), 1 and 3 minutes during the recovery from the squat exercise test, and for the Ruffier-Dickson Index as defined in the method section. a, Significant time x group interaction. b, Significant main effect of time. c, Significant main effect of group. d, Significant difference between Step-App group and Super-CF group. e, Significant difference between CF-App and Super-CF group.

Maximal oxygen uptake estimated by the Ebbeling treadmill walk test did not show any significant time × group interaction (F(1;30)=0.543, *P*=.59). However, there were significant main effects of time (F(1;30)=17.451, *P*<.001) and of group (F(1;30)=5.380, *P*<.01). Although a significant main effect of time associated with positive deltas in all groups showed an overall improvement in CRF levels in all three groups (Step-App: +0.95 mL/kg/min; CF-App: +1.70 mL/kg/min; and Super-CF: +1.85 mL/kg/min), post-hoc analysis showed a significant difference between the Step-App group when compared with the Super-CF group (*P*<.001) (Figure 5 A).

A significant time × group interaction was found in week mean step counts (F(2;60)=4.903, *P*<.01). Follow-up analysis showed a significant difference at week 1 and 2 between CF-App and Super-CF group (*P*<.01 and *P*<.05) (Figure 5 B). In detail, the Step-App group had a baseline mean step count of 8512 steps/day, 9438 steps/day at week 1, and 9246 steps/day at week 2; the CF-App group: 6808 steps/day at baseline, 7534 steps/day at week 1; and 7775 steps/day at week 2; finally the Super-CF group had a baseline mean step count of 6479 steps/day, 10,005 steps/day at week 1; and 9763 steps/day at week 2. mBeats expressed as a percentage of the participants’ weekly target showed a significant time × group interaction (F(3.231;48.463)=7.909, *P*<.001). Post-hoc analysis underlined significant differences between the Step-App group and the Super-CF group during week 1 (*P*=.045) and week 2 (*P*=.03) (Figure 5 C). When only Step-App and CF-App were compared just one significant interaction was found. This was for mBeats (F(1.580;30,019)=8.933, *P*<.001), showing a simple main effect of time in higher mBeats at week 1 and 2 in the CF-App (*P*<.001). There was a significant main effect of time in Ebbeling predicted CRF (F(1;19)=8.814, *P*<.001). Steps counts showed two trends, one of a main effect of time toward an increase (F(2;38)=2.994, *P*=.065) and the second one of a main effect of group (F(2;38)=3.998, *P*=.060), but no interaction.

### Heart Rate Outputs and Ruffier-Dickson Index

HR rest did not show a time × group interaction (F(1;30)=2.169, *P*=.13). However, main effects of time (F(1;30)=12.310, *P*<.001) and of group (F(1;30)=11.132, *P*<.001) were observed. In particular the post-pre HR rest difference was significantly lower (*P*<.001) in the Super-CF group (-7.84 bpm); compared with Step-App group (-2.74 bpm) and the CF-App group (-3.33 bpm) (Figure 5 D). The peak HR during the squat test showed no time × group interaction (F(1;30)=1.508, *P*=.36). However, there was a significant main effect of time (F(1;30)=6.201, *P*=.02) but not between groups (F(1;30)=1.683, *P*=.20). All three groups recorded lower HR peak due to the three different interventions (Step-App: 119.4 bpm; CF-App: 122.2 bpm; Super-CF: 127.7 bpm ) (Figure 5 D).

HR recovery after 1 and 3 minutes did not show significant time × group interactions (F(1;30)=1.368, *P*=.27; F(1;30)=0.832, *P*=.49). HR measured after 1 minute revealed main effects within (F(1;30)=9.318, *P*<.01) and between groups (F(1;30)=16.798, *P*<.001). The Super-CF group had a significantly lower HR (-11.71 bpm) than both the Step-App (-5.01 bpm; *P*<.001) and the CF-App (-3.42 bpm; *P*<.001) groups. Also HR recovery measured after 3 minutes showed a main effect of group (F(1;30)=7.989, *P*<.001). After 3 minutes, the HR in the Super-CF group (-10.17 bpm) was significantly lower than the one in the Step-App (-3.31 bpm; *P*<.001) and the CF-App group (-4.87 bpm; *P*<.01). Conversely, no within group differences were observed (F(1;30)=1.634, *P*=.21).

In order to confirm the validity of the RDI as CRF index, we correlated it with VO_2_ max estimated using baseline values. A negative significant correlation was present between RDI and the estimated VO_2_ max (r=-0.46, *P*<.01). We then have used this as an additional marker of CRF. RDI did not show any time × group interaction (F(1;30)=1.148, *P*=.25), but significant main effects of time (F(1;30)=6.679, *P*=.02) and group (F(1;30)=3.374, *P*=.03). The RDI was significantly lower in the Super-CF group than the Step-App (*P*<.05) and the CF-App (*P*<.01) groups (Figure 5 D).

When only Step-App and CF-App were compared, only two significant main effects of time were found. One for HR after 1 (F(1;19)=4.619, *P*<.05) and the other for HR after 3 minutes (F(1;19)=4.289, *P*<.05).

## Discussion

### Principal Findings

Our research showed that a scientifically endorsed program to increase CRF, in line with ACSM's guidelines, implemented on smartphone in an easy to use and always-accessible app can improve fitness, and other health-related parameters. According to the treadmill walk test, CRF increased in all three groups not showing any interaction between the groups. Although, the weekly mean number of steps walked by the CF-App group did not increase drastically from baseline levels (6808 steps/day at baseline; 7534 steps/day week 1; and 7775 steps/day week 2), and it may seem that participants in the CF-App group were more efficient than those in the other two groups in improving their CRF levels; yet no interaction and no main effect of group were found between the Step-App and the CF-App groups indicating that steps/day did not differ that much among the two App groups. The mBeats for the CF-App group did increase significantly from 43.5% of the mBeats target at baseline, 113.7% at week 1 and 105.5% at week 2, whereas mBeats in the Step-App group did not increase (ie, 60% of the hypothetical target mBeats). Although the Super-CF group achieved a higher mBeats level (ie, approximately 170% of the target mBeats), the participants of this group have done that by walking the highest number of steps per day. Most probably because these participants trained at the gym under the supervision of a personal trainer, three to four times a week. These results seem to suggest that intensity as well as volume training delivered by means of an easy to use mobile app accessible at any time, may be an efficient alternative to attending fitness classes. Although programs targeting steps can be a useful tool for sedentary people with a very low CRF level [7], this may be inadequate for people with higher baseline PA levels and CRF; this is particularly true if stepping rate is not high enough (eg, >150 steps/min) [36].

Resting blood pressure results showed a small but significant decrease in SBP in the two intensity groups (CF-App and Super-CF), but not in the volume group (Step-App). DBP decreased in all three groups. Previous studies have found that both volume and intensity training are able to reduce blood pressure in hypertensive people [37]. It is important to point out that our sample was composed of normotensive individuals.

Resting as well as recovery HR did decrease, as expected [37], in all three groups because of both volume (ie, steps) and intensity (ie, HR) interventions. However, the Super-CF group showed the largest effect. Accordingly, peak heart rate during a squat test showed improvements mainly in the Super-CF groups.

### Limitations

Our study had a number of limitations. Cardiorespiratory fitness was only indirectly estimated by using a treadmill submaximal test and a squat test [30,31]. The first has been shown to have high accuracy and repeatability [38], while the second was used because it has the advantage not to require any specific laboratory equipment except for a HR monitor, a stopwatch, and a metronome. The latter two elements are easy to implement on a smartphone, which would make the squat test ideal for self-testing. The good correlation between the squat test and the treadmill test indicates that a squat-based self-assessment test could be implemented to such a CF-App, in order to track progress. This correlation also confirmed the evaluation of Sartor et al [38], whom suggested the use a squat test, for the home settings; still a further thorough validation of this test is required.

### Conclusions

Our CF-App was built taking into consideration the user experience feedback. As shown in the Methods section an additional qualitative study was conducted on 18 people to improve the look and feel and the experience flow of our software app. Qualitative reports confirmed the high relevance and acceptance of our app. However, it could be improved by providing educational, interpretational, and motivational messages. Ultimately, it is important for people who have a low PA level and a low CRF to start doing something to improve their lifestyle behavior. Adhering one to one to guidelines and recommendations can be overwhelming for most people [17]. By implementing those guidelines in a portable device and providing straightforward feedback on daily as well as weekly progress, without penalizing users when goals are not strictly met, could be an interesting way forward in cardiorespiratory health promotion and CVD prevention. Step-counting–based PA programs are a good starting point for sedentary people as long as these are designed to incentivize a breaker pattern [39].

Another important aspect of our research was to encourage people to take any occasion throughout the day to engage in moderate to vigorous PA. For the less fit people this meant taking the stairs more often, cycling to work at a faster pace, or brisk walking during the lunch break instead of having a stroll. By no doubts motivation is key, and people with low readiness to change and self-efficacy will still struggle to adopt an active lifestyle. In the current study, given 100 as the maximal motivation to exercise, we had moderate levels in self-efficacy in all three groups, averaging approximately 54.3 ± 9.1. Yet all groups showed good short-term adherence throughout the two intervention weeks, still longer-term adherence, which is the hardest to achieve, remains to be investigated.

For logistics reasons, participants were not randomly assigned to the Super-CF group. This has resulted in a baseline difference in CRF. However, this group was mainly used as a quality check of the intervention, to control for the main effect of time. This study, as mentioned above, was also kept rather short, only 2 weeks of intervention. We have shown in the past that sedentary people can improve their CRF when undergoing a vigorous but short training program [40]. The choice to keep the study short was mainly dictated by the necessity to have high adherence to prove the principle that CRF can be modified by an unsupervised app-supported program. However, this did not enable us to investigate long-term adherence. Consequently, future studies should examine long-term adherence for such an app-based program and determine how to implement guidelines into flexible exercise prescription, which can be adapted according to users’ needs, without jeopardizing effectiveness.

In conclusion, a 10,000 steps/day target-based app improved CRF similar to an ACSM guidelines-based program whether it was implemented on a mobile app or in supervised gym sessions. Moreover, HR-based training improved CRF in equal measure as a steps-based training, but with a higher number of heart beats in a training zone for a similar number of steps/day than a Step-based training.

## Abbreviations

ACSM: American College of Sports Medicine
ANOVA: analysis of the variance
BMI: body mass index
CF-App: cardio-fitness app
CRF: cardiorespiratory fitness
CSUQ: computer system usability questionnaire
CVD: cardiovascular disease
DBP: diastolic blood pressures
HR: heart rate
HRR: heart rate reserve
PA: physical activity
RDI: Ruffier-Dickson index
Step-App: step-based app
Super-CF: Supervised Cardio-Fitness
SBP: systolic blood pressures
